# Transactions of the Medical and Physical Society of Calcutta

**Published:** 1829-10-01

**Authors:** 


### Transactions of the Medical and Physical Society of Calcutta. Vols. II, and III. Calcutta, 1826-7.

### Art. I.—Observations on Diseases of the Spleen, particularly on the Vascular Engorgement of that Organ common in Bengal. By *W. Twining*, Esq.

To acquire a knowledge of the diseases of an organ, and of the influence of such diseases on the general system, it is not absolutely necessary to know the real function of that organ in health. We know what bronchocele is, and we can often cure it, without being able to ascertain either the use of the thyroid gland or the cause of its enlargement. The function of the spleen is a matter of mere speculation; but its diseases are of very serious importance, especially in warm climates, and even in the marshy districts of our own country. It is in India, however, that splenic diseases are seen on a large scale, and as Mr. Twining has had ample opportunities of investigating the subject, a brief account of his paper may not be uninteresting.

"The vascular engorgement of the spleen to which I allude, is in this country attended with a peculiar state of constitution, unequal to bear the action of mercury, without great hazard of inducing alarming debility, and depression of vital power, with tendency to sloughing of the lips, cheeks, and gums, which when once commenced, does frequently, or I may rather say, generally, terminate fatally. The state of constitution to which I now allude, appears to depend on some condition of the solids, or fluids, or of the vital actions, superadded to the state that is generally denominated debility." 351.

This disease is frequently seen idiopathic in children, arising from damp climate, want of exercise, bad clothing and feeding, &c. though it is most commonly a sequela of fever:—"In fact it is the most invariable consequence of acute diseases among children of weak constitutions in Bengal." A similar enlargement, however, takes place in the spleen of adults, in consequence of various debilitating diseases—especially intermittent and remittent fevers.*

"Vascular engorgement of the spleen is characterised by a pale, and often

---

* Of the European troops who returned from Arracan, a great number laboured under the disease which is now to be described.

lurid or cadaverous countenance ; the tunica albuginea of the eye has a pale blue or pearly appearance, similar to that observed in hectic patients ; the skin is generally dry, and there is some morbid heat of surface, especially over the belly. There is tumour and tenderness in the region of the spleen, with tension at the epigastrium, which often extends across to both hypochondria. The patient suffers from thirst, though his tongue is generally clean and moist ; sometimes it is of a pale and glossy hue, having its surface marked with deep fissures : more rarely it is of a morbid red colour. The stools are generally scanty and frequent, with tendency to constipation ; urine pale and copious. The pulse is quick, and occasionally hard ; appetite indifferent and digestion imperfect. The debility is greater than would be at first apprehended from observing the size and appearance of the patient.

"Agues frequently return while these symptoms remain, and the hot stage is protracted, and generally peculiarly distressing. This I should denominate the *irritative stage* of tumid spleen : and I have reason to believe, that its peculiar combination of constitutional irritation with obscure pyrexia, and general debility, are most frequently found to exist after remittent and intermittent fevers of low and damp situations, where there has been a premature use of wine, and undue quantity of stimulating diet.

"This febrile or irritative stage occurs less frequently than the simple congestion, in which the tumefaction of the spleen is not only greater, but more distinct, because there is less general elastic tension of the belly. There is less tenderness on pressing the spleen. With this condition of the local disease, we find that the pulse is low and weak, face of a pale and lurid colour, tongue clean and moist, skin cool with a peculiar dry feel, like a thin satin loosely laid over the soft cellular structure : there is an hectic eye, and pale urine as in the irritative stage, but less of pyrexia. There is generally more appetite, but assimilation is imperfectly performed, and nutritious food does not restore the strength. Agues relapse more rarely than in the irritative condition, except at the lunar phases, or when the bowels become obstructed, and the cold stage is most distressing from the great distension of the spleen. If this were denominated the *congestive state* of tumid spleen, it might sufficiently indicate the local disorder and corresponding constitutional derangement. This is the state which Dr. Pemberton alludes to, when he speaks of the swelling of the substance of the spleen, unattended by inflammation. Page 71, see also Morgagni, Epist. 36. Art. 17, and 23.

"In both the above described stages of diseased spleen, the respiration is low, languid, and imperfect, the lungs being but partially filled with air ; the stools are mostly of a dark colour, which with the lurid cadaverous countenance, and pale lips, indicate the imperfect performance of those functions whereby the blood is decarbonised. It would not appear correct to ascribe the dark colour of the stools in these cases to disordered bile : repeated and continued attention to this subject leads me to believe, that in the disease now treated of, the whole system is suffering ; and there is a feeble effort of nature to compensate for the inefficient action of the lungs and skin, the great intestines secreting carbon, which gives rise to the dark coloured stools." 357.

In females, amenorrhœa is an early attendant on the disease. The muscular debility, unattended with corresponding emaciation, and the great despondency of mind, may probably be connected with the decarbonized state of the blood in this disease.

"Either the irritative or the congestive stages of disease before described, are occasionally followed by the *indolent tumid spleen* which is attended with little or no pain on pressing over that organ : while the spleen, though not exceedingly large, is often very hard, and its extent well defined. There is less appearance of debility, more appetite for food, and digestion is more perfectly performed than in the two foregoing forms of disease. In this indolent stage, there is often a dry cough, attended with pain in the left side of the chest, occasionally shooting to the centre of the scapula, or to the shoulder. The use of mercury, though not to be recommended, is less hazardous in this state of disease, which is always obstinate, and very difficult to cure." 358.

The progress of the malady is more or less rapid according to the injury which the constitution has sustained from climate, fevers, &c. The disease often runs its course rapidly, death being preceded by dysentery, dropsy, or hæmoptysis. Intractable diarrhœa is the most common precursor of death in Bengal. From an attentive consideration of the causes and the phenomena, our author thinks it would not be any violation of propriety, if the disease in question were denominated "*the endemic cachexia of those tropical countries that are subject to paludal exhalations.*"

The full detail of constitutional symptoms is considered necessary by our author, in order to put practitioners on their guard against mercury, the employment of which, in such constitutions, often induces gangrene of the mouth.

"The treatment generally found useful in that modification of enlarged spleen which is most frequent in Bengal, is a steady perseverance in a course of purgative medicines, combined with bitters and some preparation of iron, which, with a mild and rather abstemious diet, will almost invariably remove recent enlargements of the spleen, when in that form which I have ventured to denominate *congestive*. The *irritative state* of tumid spleen, which is attended with more decided pyrexia, and more morbid sensibility of the organ on pressure; requires the application of leeches to the side, but sometimes V. S. is preferable; and when the febrile symptoms are more urgent, I have thought the cure proceeded more favourably when the patients were purged two or three days by the Pulv. Jalap. Comp. before the sulphate of iron was administered with purgatives and bitters." 362.

A great uniformity of morbid appearances, *post mortem*, being found, not many dissections have been reported, as indeed it would be only a useless repetition of the same thing. The following may be considered as a resumée of the pathology.

"Among the morbid appearances which are most frequent, we observe, 1st. A soft enlargement, resembling a great clot of blood, wrapt in a thin membrane; this varies in colour from black to brown or blue; and when we attempt to lift the spleen, the fingers are thrust through the membrane, and the organ breaks down in the hands, becoming a putrid gore. 2d, Next in frequency, we find opacities of the peritoneal surface from lymphatic deposit thrown out during superficial inflammation; or possibly only the effect of repeated over distension. 3d. Adhesions of the peritoneal coat of the spleen to contiguous viscera, which adhesions are by no means a general result of tumid spleen in Bengal. 4th, In a few old cases, we find a more indurated friable spleen, that breaks when handled without much force, like a piece of old moist cheese. 5th, And still more rare, is the cartilaginous lumpy hardness to which we give the name of schirrus.

"Besides the above appearances of disease, I have sometimes met with an uniform pale white or milky colour of the peritoneal coat of the spleen, in patients who had been long subject to agues: the substance of the spleen being soft and flexible, but the peritoneal coat more than usually tough, and wrinkled, like a thin bladder that had been dried, and afterwards wet in hot water.

"In examining the bodies of patients who have suffered spleen disease for a long time, and are carried off by a purging; numerous small ulcers are found on the internal membrane of the great intestines, while the peritoneal coat appears either quite healthy, or paler than usual." 363.

A great number of cases are detailed by the author; but we do not deem it necessary to analyze more than the descriptive, narrative, and didactic portions of the paper. Mr. Twining informs us that he has given the tincture of iodine in six cases of chronic tumid spleen, without the least advantage. Enlargement of this organ being a very common disease among the natives of Bengal, the remedies employed by them are not unworthy of notice. Their general plan is to apply the actual cautery over the region of the spleen.

"Slight incisions are first made through the skin with a lancet, (five or seven are most common, though in bad cases 20 incisions are made:) a needle is then inserted at each of these cuts in the skin, and thrust about half an inch obliquely into the integuments or muscles of the belly; after which a heated iron instrument, the size of a large nail, is applied to each of the incisions. The patient is ordered a low diet, chiefly of fish, curry, and rice, with injunctions to take but little drink, and to eat no butter, milk, oil, or fat. A mixture of aloes, garlic, and vinegar is given daily; and about a grain of kussees (common sulphate of iron, as it is sold in the bazar) is given with each dose. The quantity of aloes is regulated so as to produce three stools in 24 hours.

"This I consider a very efficacious plan of treatment, if used in recent cases; and I have seen numbers of persons with the marks of having been severely cauterized over the left hypochondre, in whom the enlargement of the spleen has entirely subsided. But there exist cases not to be cured by the utmost perseverance in this treatment; for in the course of last winter, several native patients were brought to my house, exceedingly emaciated, and suffering from indolent enlargement of the spleen: which had acquired such size, that I considered the disease incurable in those reduced constitutions. These patients all had very numerous scars from the cautery, and said they had taken the aloetic remedy with vinegar; but received no benefit from persisting in its use, for a very long period. The usual Bengal prescription is

| | | | |
|---|---|---|---|
| Garlic, seven tolahs, or about | - - - - - | 1253 | grains. |
| Aloes, two tolahs, " | - - - - - | 358 | ditto. |
| Kussees (or sulphate of iron) 1½ ditto, or | - | 268½ | ditto. |

Mixed, and made into 57 bolusses, one for an adult every morning, ½ to a woman, and ¼ to a child from two to twelve years of age. This medicine is ordered to be taken in a small quantity of confection of roses." 400.

Mr. T. has often employed issues over the region of the spleen, with benefit in chronic cases, sometimes using the moxa, and occasionally the heated iron. "The effects of the latter appeared to be far superior to blisters in chronic cases." European patients asserted that they suffered less from the actual cautery than from the moxa.

Mr. Twining's paper is followed by another very short one by Mr. Wilson on "the native treatment of diseased spleen." This needs but little notice. The following passage shews that the Hindoo pathology is not very different from the fashionable doctrines of the day in this country.

"The causes of all visceral complaints are declared by the Hindoo physicians to be indigestion, occasioning accumulated fæces, and visceral obstruction. In this case, the belly swells, with pain and heat, the evacuations are scanty, there is much flatulence and uneasiness, the superficial vessels become enlarged, and assume a retiform appearance, the appetite is lost, there is great prostration of strength, and much languor, with vertigo and fainting, the colour leaves the lips and the palate, and the person looks like a *preta* or ghost. When the biliary humour is especially affected, the pain, heat, and fever are more intense, and the skin is of yellow tint; and when the phlegmatic humour is chiefly vitiated, the pallor with lassitude and drowsiness are most remarkable." 414.

The Hindoo treatment consists chiefly in purgation with castor oil and cow's milk, and a number of herbs in decoction, probably of little efficacy. When the spleen becomes enlarged, and much pain is felt, blood is taken from the left arm, the spleen being rubbed at the same time to promote the flow of blood. If these measures fail, the actual cautery is employed in one of two ways.

"In the milder application, the part is washed with warm water, and the number of places to be burned are marked by so many drops of oil. These are covered with small pieces of young plantain leaves, and a wet cloth is laid over the whole. A piece of lighted stick, of the wood of the Jujube tree, is then applied to each spot. No pain is felt upon the contact of the burning wood, but on removing the cloth and leaf, a vesicle is formed, which ends in an ulcer.

"When this is unavailing, recourse is had to the second operation, in which iron needles, heated red hot, are plunged into the side above the spleen in various places according to the discretion of the operator, usually not fewer than seven, nor more than twenty. It seems likely that they penetrate its substance. The needles are made to penetrate to a considerable depth, or according to the native idea, through the spleen. The operation is attended with violent pain, but is said rarely to fail of success." 418.

### Art. 2. Arracan; its Medical Topography, and the Diseases which affected our Troops in that Quarter. By Mr. *R. N. Burnard.*

A great portion of the third volume of the Calcutta Medical and Physical Transactions is occupied with details of the medical topography and diseases of Arracan and Rangoon. The difficulties and dangers into which our troops, as well as our medical practitioners, were thrown, during those disastrous, though triumphant expeditions, "through jungle, brake, and fen," will long be remembered by those who participated in them;—but we fear that they will be too faintly portrayed on the page of medical history, in consequence of that cold apathy which freezes the routine practitioner's breast, on all occasions where his daily avocations are not directly concerned! The medical topography and the prevailing diseases, however, of all countries, are interesting, no matter how remotely situated, since these portions of medical history shew the connexion between causes and effects on a large scale.

Arracan is now become more than usually interesting to Englishmen, in consequence of the additional spread of our conquests in that direction. If it has been the theatre of glory to our warriors, it has also been their grave! Few situations have probably been ever occupied by an Indian army so productive of disease, misery, and mortality, as Arracan. The sickness there was not the casual visitation of an epidemic, like the cholera, but was evidently *endemic*—dependent on local causes, and those causes visible to every one who made medical topography his study.

The military operations against Arracan having been completely successful, there were no depressing passions in action at first; but these were soon after called forth by "the hourly sight of death and suffering among their comrades," and the prospect of a long residence in the same situation. The following is a concise topographical description of this pestilential spot.

"It is situated in Lat. 20° 35′ N. Long. 93° 32′ E. distant in a direct line from the sea about fifty miles, but having a navigable river running close up to it, and then dividing into several smaller branches, which flow through the town in every direction. The average rise of the tide is about eight feet at the town; but spring tides rise higher, and consequently cover a considerable space of ground, which at other periods is free from the flux and reflux. The banks of the stream generally through the town, are low, and covered with sedge, coarse grass, and a few bushes, serving as a receptacle for filth of every description. The stream itself is made the receptacle of dead bodies, many of which, in the most offensive states of putridity, are constantly to be seen, at the different points where an obstruction is offered to their progress towards the main stream. Proceeding towards the sea; below the town, the banks rise very little above the level of the water, are covered with jungle, and intersected in every direction by smaller streams, forming a complete and impassible Sunderbund. Arracan bears about N. E. from the mouth of the river, and three distinct and parallel ranges of hills are visible from it: the town itself is situated, with respect to the general line of the first range nearly as the apex of a triangle to its base; but from the number of insulated hills and slight curvatures in the range, it appears nearly embayed in a recess of the hills. The height of the highest hill in the first or Arracan range is 550 feet, of the second and third ranges respectively estimated at 2000 and 4000 feet. The hills, generally speaking, are abrupt, and many of them insulated; they are covered with jungle, and have many channels cut in them by the violence of the periodical rains. The waters thus collected in the hollows between the bases of the hills, either meander amongst them till received into some channel which conveys them to the river, or they, for want of an outlead, lie till absorbed or evaporated. About a quarter of a mile from the N. W. angle of the fort of Arracan is a large lake, extending in this meandering way to a distance of several miles amongst the hills: this lake is full of weeds, and its banks low and marshy; the average depth of water I think about eight feet, but very unequal. In every situation where water can lie during the heavy rains, it collects, and forms a swamp, capable, when the drying process commences, of giving out miasmata of a destructive nature. The hills consist principally of schistus; no granite (as far as I know) has been observed; the soil is luxuriantly rich, and the mango and other trees attain great perfection in the vallies: on the hills the jungle is principally underwood, but on the higher ranges bamboos are said to abound. Beyond the highest range of hills, the country is described as being a fine open champaign with navigable streams, and a much less quantity of rain, and is stated to be particularly healthy, a statement which meets confirmation from the immunity enjoyed by our troops during their late residence on the banks of the Irrawuddee. It is also said, that such of the Mughs as could afford it, and their Burmese conquerors always withdrew to the banks of that river during the rainy season." "From whichever quarter the wind may blow, it must, before reaching Arracan, blow over a considerable extent of ground in almost every variety of condition: the consequence is, that every gale comes impregnated with the elements of disease. The grand difference between Arracan and the Delta of the Ganges consists in the want of a *general* and abundant annual inundation. In Arracan no river of magnitude sufficient to overflow the low lands exists, and a sufficient quantity of water either falls from the clouds, is furnished by high tides, or descends from the hills, to keep up a perpetual renovation of swamp, or rather to place the soil in that state of approximation to dryness which I believe to be essential to the extrication of marsh miasmata. In Bengal, as is known to every man of observation, the period at which the endemic fever chiefly prevails, is when the inundation has subsided, and the surface, then approaching to a dry state, is exposed to a powerful sun; and the fever thus produced is particularly severe in situations where jungle of any kind is within the sphere of the inundation. The high state of cultivation in Bengal has doubtless done much to ameliorate the climate; and in the rice grounds, the more extensive the inundation the less is the insalubrity subsequent. This extent of inundation, which absorbs as it were the morbific principle, and distributes it over so large a space as to render it comparatively inert, is wanting in Arracan; and in every place similarly situated, there must be marsh in every stage of moisture and approaching to dryness." 31.

Particular spots were found to be more unhealthy than others—and these corresponded exactly with their greater exposure to streams of air coming over malarious localities. It would be difficult to imagine a place more copiously supplied with the elements of disease than that which our author has sketched out in the foregoing extract;—and the reader will not be unprepared for the announcement of a severe visitation of sickness among our troops. The general characters of the fever here prevalent did vary essentially from those of the common endemics of other parts of India.

"The attack of the fever was sometimes sudden; but in general, loss of appetite, lassitude, slight head-ache, and nausea, preceded the developement of the febrile symptoms: a sense of coldness was generally felt, but did not, except in a few cases amount to distinct rigor; and when shivering did take place, it might be looked on as the commencement of a disease which more speedily and decidedly assumed the intermittent type. To the symptoms above mentioned, in a few hours generally succeeded heat of skin, head-ache, with a sensation of tightness and weight over the eyes, flushed countenance, full and quick pulse, white and dry tongue, anxiety, restlessness, great thirst and disposition to vomit: in some instances, the irritability of the stomach so great as to reject every thing. The bowels sometimes costive; but more commonly small, scanty, and vitiated stools were passed, with some griping and heat, every five or six hours: no feeling of relief was produced by these evacuations, the patient complaining that they were unsatisfactory, and of a sensation of a load still remaining. What was rejected by vomiting usually consisted of mucus, and the fluids drank: very little bile, in the greater number of cases, passed into the stomach. Whether produced by deficient secretion, or by a spasmodic constriction of the ducts, bile was in many instances absorbed, and conveyed into the circulation, tinging every part of a bright yellow, giving to the fever the distinctive character of the endemic of the West Indies: few cases in which this was decidedly marked terminated in recovery. A different kind of yellowness, more intermixed with a sallow muddy tint, and by no means of so deep a hue, was visible in some of the febrile cases; but as it only occurred in those of longer duration, and where there was reason to suspect derangement either in the functions or structure of the liver, I conceived it to be more properly a sequela than a symptom of the fever. In most cases where the fever from the commencement shewed a disposition to intermit, it was by assuming the double tertian type, but this passed not unfrequently into the remittent. Where the event was fatal, it was usually by lesion of some important organ, and that induced gradually, not by the immediate and active operation of febrile excitement, and the most common mode was by serous effusion in the brain, or between its membranes." 35.

"Dysentery and diarrhœa were the diseases which proved most fatal, not in their acute form, but ensuing as a consequence of fever; and their ravages, particularly amongst the native troops, were very great. The usual course was for a man to be admitted into hospital with fever, which after some time is succeeded by purging: the fever then goes off, and after a time returns; and the purging ceases, in this manner alternating perhaps for several months, when anasarcous swellings take place, extreme emaciation, and the patient dies perfectly exhausted. The purging is of various characters, sometimes light yellow evacuations, with a trifling degree of consistence, and not attended with much griping; others consist of this yellow discharge, intermixed with green bilious colluvies, and attended at times with severe griping: from some the discharges are merely dirty water, or like the washings of raw meat; and from some the intestinal mucus is discharged in considerable quantity, mixed with blood, and occasioning severe pain. Prolapsus of the rectum takes place in some of the most severe cases; and I have seen a considerable protrusion of the intestine, distended as if infiltrated by serum, and with points of blood oozing from its mucous surface without ulceration. Amongst the evacuations streaks of pus were occasionally to be observed; but the more frequent forms were the sanious, and the mixed green and yellow discharges. In the latter stages of some of these cases, the irritation extending to the bladder, gave rise to dysury. The debility continuing to increase, dropsical swellings make their appearance, sometimes generally, sometimes locally, but most frequently commencing by anasarca of the feet and legs, which gradually extends to the body. Depositions of water in the abdomen are less frequent; but I have used the paracentesis in some of these cases, more with a view of relieving the patient from the load and oppression caused by the fluid, than with a hope of ultimate success. Few indeed are the cases of this chronic dysentery, in which recovery takes place after the appearance of dropsical swellings. In the cases where the collection has been in the abdomen, this discharge of the fluid generally enables the examiner to discover enlargement of the liver or spleen, or perhaps both." 40.

The causes of the above and many other forms of disease were obvious enough. Febrific miasmata, exposure to the weather, and especially to the rains, and intoxication, were the chief items in the etiological catalogue.

"In the treatment of the disease, much depended on the period at which it was instituted. During its formation, the application of decided measures frequently succeeded in rendering what would otherwise have been a remittent, intermittent; or at least in making the first remissions so distinct, that the disease was speedily disposed to assume the intermitting form: that it rarely happened that any measures at once cut short the disease, was to be expected, the patient still continuing within reach of the exciting cause, which had produced the effects for which remedial measures were instituted, and which was not likely to operate with the less force because the system was already under its influence. In proportion also to the suddenness and inflammatory appearances of the attack, were decisive measures more plainly indicated at the commencement, and more frequently successful. The mode of treatment which I was in the habit of adopting, when called to a patient laboring under the first symptoms of an attack of this fever, particularly if marks of determination to an important organ existed, was immediately to open a vein, and suffer the blood to flow *ad deliquium*, or the *relief of the symptoms*. One of these occurrences, and not the quantity of blood taken, is the criterion by which the effects of the measure must be estimated: having a determined object in view, viz. to create a change in the existing state of things, and if possible to break the diseased associations at once, the remedy must be applied decidedly, and carried to the extent of producing an effect on the system afterwards to be maintained by other measures. From twenty-five to forty ounces is the quantity usually required; but I had no hesitation in going beyond this when circumstances demanded it. The feeling of weakness in these cases, especially *at the commencement*, is not real; it arises from oppression, and when that oppression is relieved, the strength is in a great measure restored. The immediate effects of a bleeding carried to the extent above mentioned, are, relief of organs laboring under oppression from congestion, an alteration in the circulation, generally followed by profuse perspiration, and a mitigation, if not altogether a solution, of the paroxysm. To maintain these favourable symptoms, when produced, other measures become necessary; and the first of these is to clear the alimentary canal by purgatives, and those liberally administered. Calomel alone, or with Cathartic extract, followed by Magnes. Sulph. in Infus. Sennæ in repeated doses, till free or even severe purging is produced, answers best for this purpose. If the head-ache, pain, and sense of tension over the eyes have been severe before the bleeding, the hair should be cut short, or shaved off, and wet cloths kept constantly applied over the forehead; whilst, to allay thirst, the patient may be allowed the free use of acid drink. Soda water is in many cases, (particularly where there is irritability of the stomach,) most grateful. After a longer or shorter interval, according to the violence of the attack and the idiosyncrasy of the patient, the symptoms returned; but if they had been actively combated at first, generally with reduced violence, and very rarely required the use of the lancet a second time; the headache, or tension and tenderness of the epigastrium, with irritability of the stomach, being best relieved by the application of leeches, and sometimes by blistering over the latter: blisters to the shaven scalp were more adapted to an advanced stage of the disease, when serous effusion was threatened in the brain or its membranes. Where there was a dry heat on the surface, and no particular marks of determination existed, no tendency to vomit, and the headache apparently depending on the general increase of the circulation, the cold affusion proved both useful and agreeable, and in the natives, few of whom required bleeding, was a most valuable remedy. These preliminary measures having been employed, or such of them as were deemed advisable, the remedy next resorted to was mercury, or rather calomel alone, or combined with camphor or antimonial powder, or both. The usual prescription was three grains of each of these substances every four or six hours; and if sufficient evacuations were not produced by it, a purgative was occasionally interposed. In one or two instances, the camphor excited an unpleasant sensation of heat in the stomach; but more frequently it appeared valuable as an auxiliary, in determining to the skin, and allaying irritability.* Where there was much disposition to vomit, I have occasionally substituted a small quantity of opium for the antimony. In a few cases, I have given calomel alone, in doses of scr. i. where I have wished to produce a rapid and decided mercurial effect: in some it has answered, but repeated disappointments have convinced me, that though safe it is not so infallible as some authors would lead us to suppose. Mercurial frictions, and dressing blistered surfaces with mercurial ointment, were also employed, but never relied on as principal remedies. Great difficulty was often experienced in procuring the mercurial impregnation, and its effects were often seen in the subsidence of the fever. Salivation is by no means essential; and when severe, is of itself a formidable disease: when mild, it is perhaps the most desirable method in which the mercurial action can be developed, as it shews, in the most obvious manner, the extent to which the operation of the remedy on the secretory system at large extends; and the variations in the salivary secretion are valuable, as marks of the approaching return or actual existence of the febrile state. That mercury, however administered, often fails to affect the system,—that having produced what is called its specific effect, fever not unfrequently continues its course to a fatal termination,—that relapses often take place whilst the system is under its influence,—and that its administration to some habits is poisonous, are facts too well known to admit of denial; but is is not from the occurrence of a few untoward events that a principle of practice is to be laid down; attentive observation, on an extensive field of experience, will shew the proper degree of importance to which the objections against

* "Since my return to our own provinces, I have received from England the Report made to the Madras Government on the fever of Coimbatore and adjoining districts, by Drs. Ainslie, Smith, and Christie, and am happy to find the ideas of those gentlemen on the subject of combining camphor with calomel so congenial with my own. 'There appears to be something in the nature of camphor, which peculiarly fits it for being given in conjunction with calomel: it mitigates in some measure the acrimony of this mineral, tends to calm the nervous system, and gives the fluids a tendency to the skin.'—*Report on the Coimbatore Fever*, Note, page 154."

the remedy are on this account entitled: and I believe I am borne out by the general experience of my professional brethren in this country, in saying, that we have no remedy of such general utility in febrile affections as Mercury. Opium was by me rarely employed, except when the fever had become decidedly intermittent, and then was given as a stimulant, at the approach of the cold fit." 57.

For the cases and meteorological tables we must refer to the volume itself. The paper in question is very creditable to the author.

